# Supplementary material for: Shortening the Lipid A Acyl Chains of Bordetella pertussis Enables Depletion of Lipopolysaccharide Endotoxic Activity
Source: Vaccines (Basel). 2020 Oct 9;8(4):594. doi: 10.3390/vaccines8040594 (PMC7712016; doi:10.3390/vaccines8040594)
Supplement: Supplementary file 1 [file vaccines-08-00594-s001.zip › supplementary--r1--ae/supplementary--fIGURE.pdf]

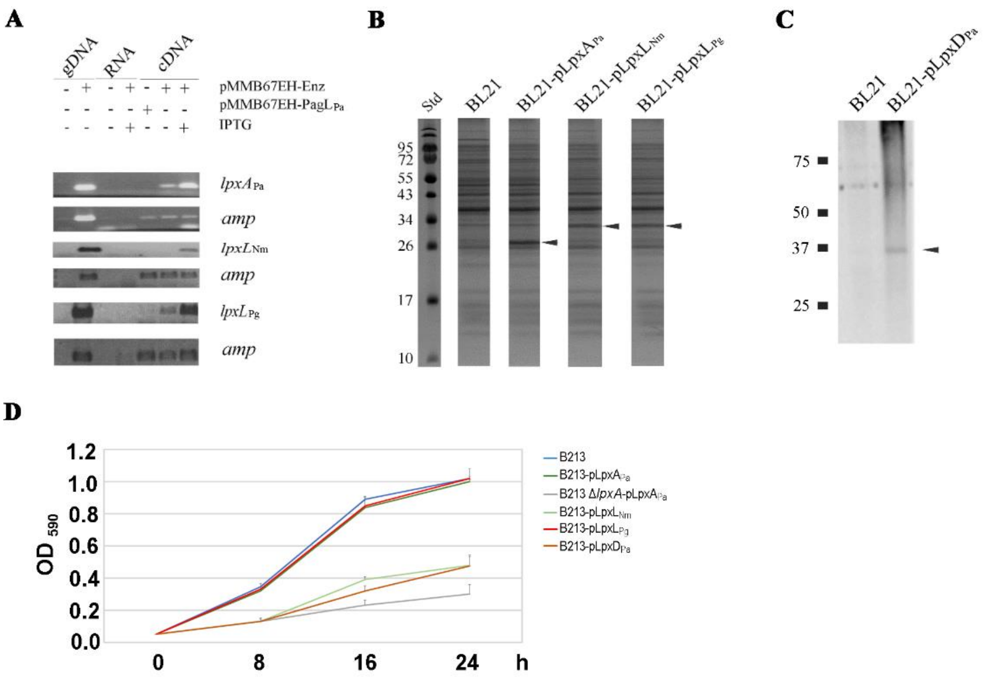

Figure S1. Expression of recombinant genes in *E. coli* BL21(DE3) and *B. pertussis* B213. (**A**) Agarose gels showing PCR products obtained with primers targeting the genes of interest or the *amp* gene encoding β-lactamase (as indicated on the right) from preparations of genomic DNA (gDNA), RNA or cDNA obtained from BL21(DE3) derivatives carrying various recombinant pMMB67EH plasmids (pMMB67EH-Enz). The strain carrying pMMB67EH-PagL$_{Pa}$, which consist of the same vector, but with an irrelevant insert (the *pagL* gene of *P. aeruginosa*), was used as a negative control to demonstrate specificity of the PCRs. Bacteria were grown in LB medium in the presence or absence of IPTG as indicated at the top. (**B**) Coomassie blue-stained gel loaded with whole-cell lysates of BL21(DE3) derivatives expressing genes for acyl transferases from plasmids as indicated at the top. The bacteria were grown in the presence of IPTG. IPTG-induced bands are indicated with arrowheads at the right. The positions of molecular mass markers (Std) are indicated in kDa at the left. Predicted molecular masses of LpxA$_{Pa}$, LpxL$_{Nm}$ and LpxL$_{Pg}$ are 28, 33.8 and 36.4 kDa, respectively. (**C**) Western blot probed with an anti-His-tag antiserum showing the production of LpxD$_{Pa}$ in BL21-pLpxD$_{Pa}$

grown with IPTG. The calculated molecular mass of the His-tagged $LpxD_{Pa}$ is 36.9 kDa. (**D**) Implication of the expression of heterologous enzymes on the growth of *B. pertussis*. The OD_{590} of cultures of B213 and derivatives expressing $lpxA_{Pa}$, $lpxL_{Nm}$, $lpxL_{Pg}$ or $lpxD_{Pa}$ from pMMB67EH plasmids after 8, 16, and 24 h of growth in Verweij medium in the presence of IPTG is shown. The starting OD_{590} was 0.05. Data are from two experiments performed in duplicate of which average and standard deviation are given.

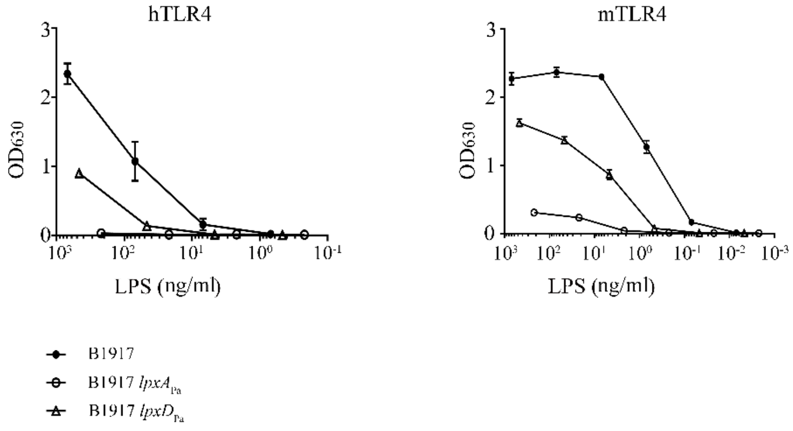

Figure S2. Bioactivity of LPS of strain B1917 and derivatives. Stimulation of HEK293-Blue cells expressing either hTLR4 or mTLR4 with purified LPS preparations of strains B1917, B1917 $lpxA_{Pa}$, and B1917 $lpxD_{Pa}$ was performed as described in the legend to Fig. 3. Graphs show data from a representative experiment of three repeats.

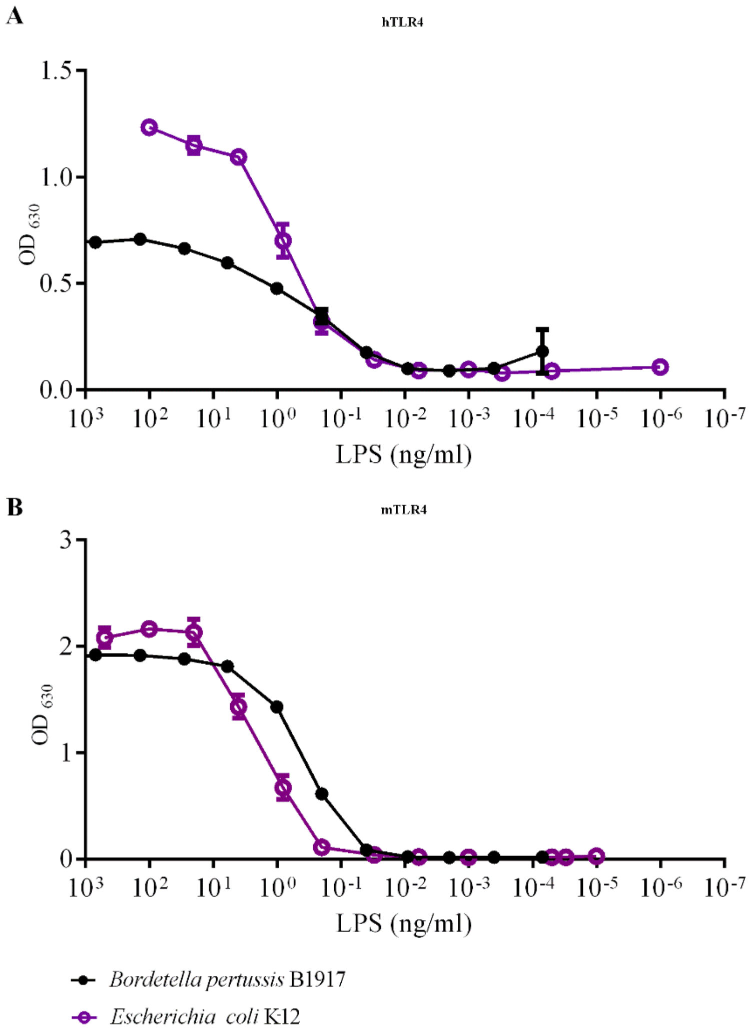

Figure S3. Comparison of the bioactivity of LPS of *B. pertussis* and *E. coli* LPS. Stimulation of HEK293-Blue cells expressing either hTLR4 (**A**) or mTLR4 (**B**) with purified LPS preparations of *B. pertussis* strain B1917 and *E. coli* K-12 was performed as described in the legend to Fig. 3. Graphs show data from a representative experiment of three repeats.

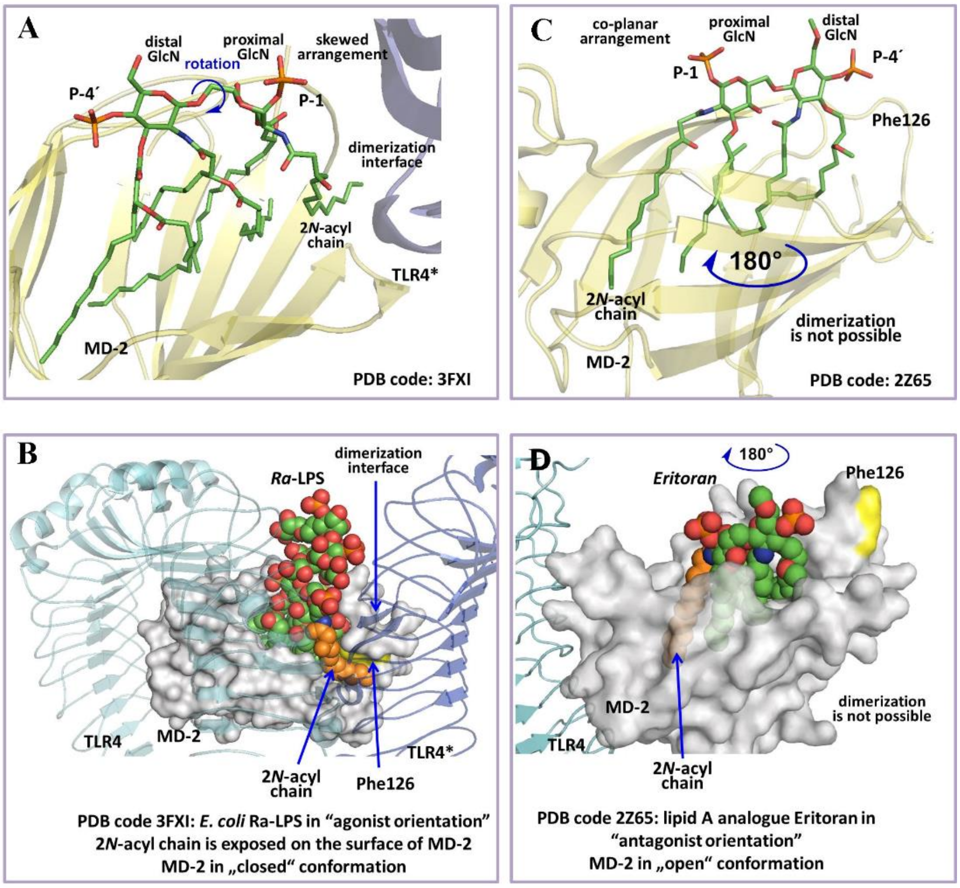

Figure S4. Binding of agonist and antagonist lipid A variants by TLR4/MD-2 complex. (**A**) hMD-2 with bound *E. coli Ra*-LPS (PDB: 3FXI); only the lipid A portion is shown for clarity. The glucosamine (GlcN) rings of the di-glucosamine backbone of lipid A are in twisted arrangement due to rotation about (1→6) glycosidic and exocyclic oxymethyl linkages. Upon binding of *E. coli* hexa-acylated lipid A by the TLR4/MD-2 complex (binding in "agonist orientation") the distal GlcN ring bearing four acyl chains is fixed in the binding pocket of MD-2, whereas the proximal glucosamine ring (which points to dimerization interface) is relocated in a tilted orientation which assists in exposure of one

acyl chain (2*N*-acyl chain) on the surface of MD-2; (**B**) hMD-2 with bound *E. coli Ra*-LPS (PDB: 3FXI and 3VQ1). The 2*N*-acyl chain is highlighted in orange. (**C**) hMD-2 with bound TLR4 antagonist Eritoran (PDB code: 2Z65). The orientation of the di-glucosamine backbone of lipid A is inverted by 180°. The two GlcN rings of the di-glucosamine backbone of lipid A are arranged in one plane (antagonist orientation); (**D**) hMD-2 with bound antagonist lipid A analogue Eritoran (PDB: 2Z65 and 2E59); all acyl chains are inserted into the binding pocket of MD-2. The 2*N*-acyl chain is highlighted in orange. Images were generated with PyMol.
