# Supplementary material for: Shortening the Lipid A Acyl Chains of Bordetella pertussis Enables Depletion of Lipopolysaccharide Endotoxic Activity
Source: Vaccines (Basel). 2020 Oct 9;8(4):594. doi: 10.3390/vaccines8040594 (PMC7712016; doi:10.3390/vaccines8040594)
Supplement: Supplementary file 1 [file vaccines-08-00594-s001.zip › supplementary--r1--ae/SUPPLEMENTARY--tABLE--AE.pdf]

Table S1. Primers used in this study

Goal PCR	Primer name	Sequence (5'->3')[a]	Remarks
Preparation of plasmids for overexpression			
pMMB67EH-LpxA$_{Pa}$	LpxA$_{Pa}$ Fw	AAGCGCGCCATATGAGTTTGATCGATCCTCG AAGCGCGCCATATGAGTTTGATCGATCCTCG	NdeI
	LpxA$_{Pa}$ Rev	AAGCGCGAAGCTTATCAGCGGGTGATGCCGCGGGTTG CGCT	HindIII
pMMB67EH-LpxL$_{Nm}$	LpxL$_{Nm}$ Fw	AACGCGCGCATATGAAATTTATATTTTTTGTACT	NdeI
	LpxL$_{Nm}$ Rev	AAGCGCGCAAGCTTTCAGTAAAAATCGGGGCTGCCTTC CG	HindIII
pMMB67EH-LpxL$_{Pg}$	LpxL$_{Pg}$ Fw	AAGCGCGCCATATGAAAGCGACACTTTCCCT	NdeI
	LpxL$_{Pg}$ Rev	AAGCGCGCAAGCTTTCATAGTTGTCGGGTATTATGCA	HindIII
pMMB67EH LpxD$_{Pa}$ -	LpxD$_{Pa}$ Fw	CGCGCGCATATGATGAGTACCTTGTCCTA	NdeI
	LpxD$_{Pa}$ Rev	GCGCGCAAGCTTTAATGATGATGATGATGATGATGAT GGCCGCCGCCCGCATCAGATGA	HindIII
Preparation of plasmids to knock out genes			
plpxL$_2$::gm	LpxL2$_{Bp}$ Fw	GCGCGCACGCGTAGGCAATTCAAGACCCGCGCGCTGA	MluI
	LpxL2$_{Bp}$ Rev	GCGCGCTCTAGACCGTTCCAGTCCACGGTAAG	XbaI
	gm Fw MluI	TCAGCACGCGTCAATTCTCGAATTGACA	MluI
	gm Rev MluI	GGTCAGACGCGTCGCCGGGAAGCCGATCTC	MluI
RT-PCR assays			
lpxL$_{Pa}$	LpxA$_{Pa}$ Fw RT	CCATTGGCGACCACAACCTG	
	LpxA$_{Pa}$ Rev RT	TGCCGAAGACCGTCACGTAG	
lpxL$_{Nm}$	LpxL$_{Nm}$ Fw RT	TGAAATCGCTGGTGCGCTAC	
	LpxL$_{Nm}$ Rev RT	ATCGGGCAGATACAGAAACG	
lpxL$_{Pg}$	LpxL$_{Pg}$ Fw RT	GTGCTACCGCTATGGATGGT	
	LpxL$_{Pg}$ Rev RT	CTATACCACGCGAACCGAAT	
amp	Amp Fw RT	CTATGTGGCGCGGTATTATC	
	Amp Rev RT	CAACTTTATCCGCCTCCATC	

Commented [M1]: Please provide the table S1 in the correct form.
