# Supplementary figures and images for: Shortening the Lipid A Acyl Chains of Bordetella pertussis Enables Depletion of Lipopolysaccharide Endotoxic Activity
Source: Vaccines (Basel). 2020 Oct 9;8(4):594. doi: 10.3390/vaccines8040594 (PMC7712016; doi:10.3390/vaccines8040594)

**Table S1.** Primers used in this study


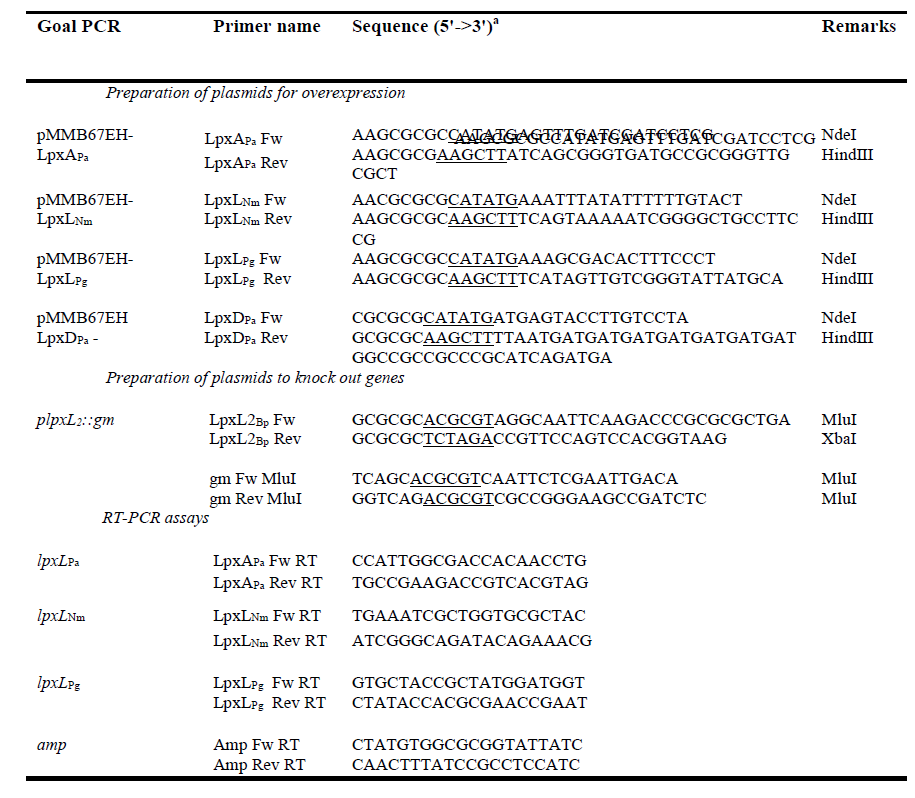

Supplement: Supplementary file 1 [file vaccines-08-00594-s001.zip › supplementary--r1--ae/SUPPLEMENTARY--tABLE--ae.docx]
